# En Bloc Resection of Bladder Tumor—Is It the Way Forward?

**DOI:** 10.3389/fsurg.2021.685506

**Published:** 2021-05-31

**Authors:** Christian Daniel Fankhauser, Marian Severin Wettstein, Luca Afferi, Nico Christian Grossmann, Hugh Mostafid

**Affiliations:** ^1^Department of Urology, Luzerner Kantonsspital, Luzern, Switzerland; ^2^Department of Urology, University of Zurich, Zurich, Switzerland; ^3^Department of Urology, Royal Surrey County Hospital, Guildford, United Kingdom

**Keywords:** trans-urethral resection of bladder tumor, bladder cancer, transurethral, urothelial atypia, urothelial cancer

## Abstract

Transurethral resection of bladder tumors (TURBT) represents the cornerstone in diagnosis and treatment of bladder cancer but recurrence is observed in up to 80% and over- or understaging with TURBT is common. A more recent development to overcome these limitations represents en-bloc resection of bladder tumors (ERBT) which offers several advantages over TURBT. In this report, we briefly review studies assessing outcomes of bladder cancer patients undergoing ERBT. Most randomized and non-randomized trial demonstrate improvement in clinical outcomes for ERBT over TURBT, however more pathological and translational studies are warranted.

## Introduction

Bladder cancer has an estimated 429,793 new diagnoses leading to 165,084 deaths per year worldwide (1) but with a globally wide variation (2). For example in the European Union, an increase by 41% from currently 124 188 up to 174 891 new cases per year is expected by the year 2035 (1). Patients with non-muscle invasive bladder cancer (NMIBC) require lifetime surveillance with cystoscopy and imaging. Due to the high recurrence-rate of up to 80% and related re-treatment, lifetime treatment costs are among the highest of all cancers, ranging from $100,000 to $200,000 per patient in the United States (3). This significant financial burden on the population and healthcare system calls for effective bladder cancer diagnostics and treatment.

Transurethral resection of bladder tumors (TURBT) represents the cornerstone in diagnosis and treatment of bladder cancer since the 1940s. Aims of TURBT include symptom relief, histological diagnosis (grade, stage, variant histology) and cure in some stages of NMIBC. Several technological advantages including bipolar TURBT have been introduced (4). However, both mono- and bipolar TURBT represent a piecemeal resection of the tumor with the risk of tumor seeding and over- and under staging because of tangential sectioning and thermal artifacts (5). This resection method ignores basic principle of oncological surgery being resection in one specimen instead of scattering malignant cells. To decrease the high recurrence rates, several new methodologies have been developed to allow en bloc removal of bladder cancers, for example in 1980 snare polypectomy a method which did not allow complete resection of the bladder base (6).

Complete en-bloc resection including the bladder base was only achieved after the introduction of en-bloc resection techniques as described in 1997 by Kawada et al. using a new arched resection loop (7). Later, the same approach but using a standard mono- or bipolar loop electrode or laser fibers have been introduced. A recent consensus agreed that any method “removing the bladder tumor in one piece” can be described as en-bloc transurethral resection of bladder tumors (ERBT) (8). All ERBT techniques have three assumed advantages. First, the histological assessment may be facilitated by en-bloc resection. Second, remaining in the same surgical plain may decrease complications. Third, avoiding tumor fragmentation may decrease the tumor spillage and improve oncological outcomes. In this report, we review studies assessing outcomes of patients with bladder cancer undergoing ERBT.

In the most recent and comprehensive systematic review and meta-analysis, Teoh et al. identified 10 randomized controlled trials comparing ERBT with TURBT (8). Limitations of those trials included low individual sample size as well as heterogeneity in outcome reporting and treatment (e.g., energy source used, postoperative management). Nevertheless, this meta-analysis represents the best available evidence and the authors concluded that compared to TURBT, ERBT has a longer operation time but shorter irrigation time and lower risk of bladder perforation. However, this meta-analysis of randomized trials was not able to show a difference regarding catheterization time, hospital stay, occurrence of obturator nerve reflex, presence of detrusor muscle in specimen or recurrence rates. Two more recent randomized trials were not included in the review. The first compared classical TURBT with ERBT using hydrodissection, both assisted by photodynamic diagnosis using hexaminolevulinate (HAL), and reported a higher percentage of presence of detrusor muscle in specimen in the en-bloc group (86 vs. 63%) (9). The second compared TURBT with holmium laser ERBT and reported a higher rate of post-operative epirubicin instillations, shorter time to catheter removal and hospital stay with a higher percentage of presence of detrusor muscle, fewer cautery artifacts and residual tumor in the pathological specimen but no difference in recurrence free survival (10).

In the same systematic review, the authors also compared the results of 22 non-randomized trials with similar findings regarding irrigation time and bladder perforation rates but discordant and more favorable results for ERBT regarding catheterization time, hospital stay, occurrence of obturator nerve reflex, presence of detrusor muscle in specimen and recurrence rates. Those findings are in line with similar studies which were published more recently and were therefore not included in the systematic review (11–14). Additionally, the authors performed a Delphi consensus and reached consensus that ERBT can be attempted in patients with <4 bladder tumors with a tumor size <3 cm. Consensus was also reached in other key areas including the statement that marking of the planned circumferential margin at least 5 mm from any visible bladder tumor before starting the resection is recommended. In order to assess the feasibility of ERBT in routine practice the same authors implemented ERBT as the primary surgical approach in all NMIBC patients with TURBT reserved as a conversion procedure in those patients where ERBT could not be completed for technical reasons. The authors found that ERBT was successfully carried out in 73% of all patients including those with large and multi-focal tumors, and 84% in patients with bladder tumors of ≤3 cm confirming that ERBT could be used as the primary approach for excising bladder tumor in the majority of NMIBC patients (15).

Whereas, direct improvements of certain clinical outcomes by ERBT are suggested by numerous clinical studies, pathological and translational studies are limited. First pathology studies suggested a higher interobserver concordance and time for analysis for ERBT specimens compared to TURBT (16) and the potential for improved sub staging in T1 disease (17). A second translational study reported a higher level of circulating tumor cells after TURBT compared to ERBT (18). Whilst the technique of ERBT has been relatively standardized irrespective of energy source used, the major current limitation of ERBT remains extraction of large en-bloc specimens, generally >3 cm. A number of techniques have been described such as the use of an Endo-catch specimen retrieval bag. Such challenges could be overcome fairly easily with collaboration from endoscopic equipment manufacturers (19).

In summary, en-bloc TURBT seem to be comparable or superior in most outcomes compared to classical TURBT and those results seem to be compelling for many urologists. A recent survey among 200 European urologists which reported that en-bloc TURBT is already the resection technique of choice in 35% of cases (20). Further development and studies of this technique are warranted.

## Author Contributions

CF and HM: manuscript drafting and literature review. LA, MW, and NG: critical revision of manuscript. All authors contributed to the article and approved the submitted version.

## Conflict of Interest

The authors declare that the research was conducted in the absence of any commercial or financial relationships that could be construed as a potential conflict of interest.
